# The chloroplast cysteine synthase complex in guard cells is critical for stress-induced stomatal closure

**DOI:** 10.1093/plphys/kiag263

**Published:** 2026-05-18

**Authors:** Sheng-Kai Sun, Rüdiger Hell, Markus Wirtz

**Affiliations:** Centre for Organismal Studies (COS), Heidelberg University, Heidelberg 69120, Germany; Centre for Organismal Studies (COS), Heidelberg University, Heidelberg 69120, Germany; Cluster of Excellence GreenRobust, Heidelberg University, Heidelberg 69120, Germany; Centre for Organismal Studies (COS), Heidelberg University, Heidelberg 69120, Germany; Cluster of Excellence GreenRobust, Heidelberg University, Heidelberg 69120, Germany

Dear Editor,

Plants must rapidly adapt to changing environmental cues such as high light and water supply. One of the fastest responses to these stresses is abscisic acid (ABA) mediated stomatal closure. Soil water limitation is known to be transduced by several mechanisms, including the xylem-mediated long-distance transport of the macronutrient sulfate (Ernst et al. 2010; Malcheska et al. 2017) and the root-borne peptide hormone CLAVATA3/EMBRYO-SURROUNDING REGION RELATED 25 (CLE25) (Takahashi et al. 2018), which both trigger ABA production in leaves. In leaves, sulfate must be reduced to sulfide and incorporated into cysteine, which triggers stomatal closure by stimulating ABA biosynthesis (Batool et al. 2018; Chen et al. 2019). In a recent study, we discovered that the dynamic formation of the chloroplast-localized cysteine synthase complex (pCSC) is crucial for ABA-driven stomatal closure in response to both soil-drying signals, sulfate and CLE25, and the high-light stress signal 12-oxo-phytodienoic acid (OPDA). Consequently, dynamic pCSC formation is critical for plants' resilience to drought and high-light stress (Müller et al. 2017; Sun et al. 2025). The pCSC is composed of serine acetyltransferase 2;1 (SERAT2;1) and *O*-acetylserine(thiol)lyase B (OAS-TL B), which activates SERAT2;1 upon complex formation to trigger cysteine synthesis. OAS, the enzymatic product of SERAT2;1, rapidly dissociates the pCSC, which allows the negative feedback inhibition of SERAT activity. Since sulfide stabilizes the CSC, both metabolites together promote the dynamic association of the pCSC towards cysteine formation (Wirtz and Hell 2006). During prolonged drought, ABA is produced in the guard cells and the vasculature. A set of cell-type-specific transporters enables rapid transport from the vasculature to the guard cells (Kuromori et al. 2018), leaving it currently unclear if pCSC formation triggered by sulfate and OPDA in guard cells is sufficient to stimulate guard cell-autonomous ABA production for stomatal closure.

In this study, we complemented the absence of OAS-TL B in the Arabidopsis *oastlb* mutant (Heeg et al. 2008) in a guard cell-specific manner. This allowed us to address the importance of guard cells acting as a sensory hub, capable of rapidly producing ABA to regulate stomatal aperture. We found that the dynamic association of the pCSC specifically in guard cells was sufficient to trigger stomatal closure in response to sulfate and CLE25. Moreover, the pCSC in guard cells was required for rapid stomatal closure in response to high-light stress and the high-light stress-associated signal OPDA.

We previously demonstrated that the absence of OAS-TL B in all cells renders plants insensitive to sulfate, CLE25, and OPDA (Sun et al. 2025). Here, we expressed *OAS-TL B* under the control of the well-characterized guard cell-specific promoter *Guard Cell 1* (*GC1*, Figure S1, Yang et al. (2008)), which causes exclusive expression of mVenus in guard cells after stable transformation of the control construct *pGC1::mVenus*. Complementation of the Arabidopsis *oastlb* mutant with guard cell-expressed OAS-TL B was confirmed in three *pGC1::OAS-TL B oastlb* lines (Fig. 1a, Table S1 and Figure S2). The *pGC1::OAS-TL B oastlb* plants were indistinguishable from the wild type and *oastlb* mutant under nonstressed conditions (Fig. 1b). Guard cell-specific expression of OAS-TL B partially rescued the accumulation of OAS in the *oastlb* mutant, although cysteine levels were similar among all these lines (Fig. 1c and d). Next, epidermal peels from these lines were generated to compare the responsiveness of the embedded stomata to external signals with that of stomata from *oastlb* and wild type plants. In contrast to the wild type control, *oastlb* failed to close the stomata in response to sulfate, CLE25 peptide, and the locally acting high-light signal OPDA. However, the expression of OAS-TL B in the guard cells fully restored the response to all three cues (Fig. 1e and Figure S3).

**Figure 1:**
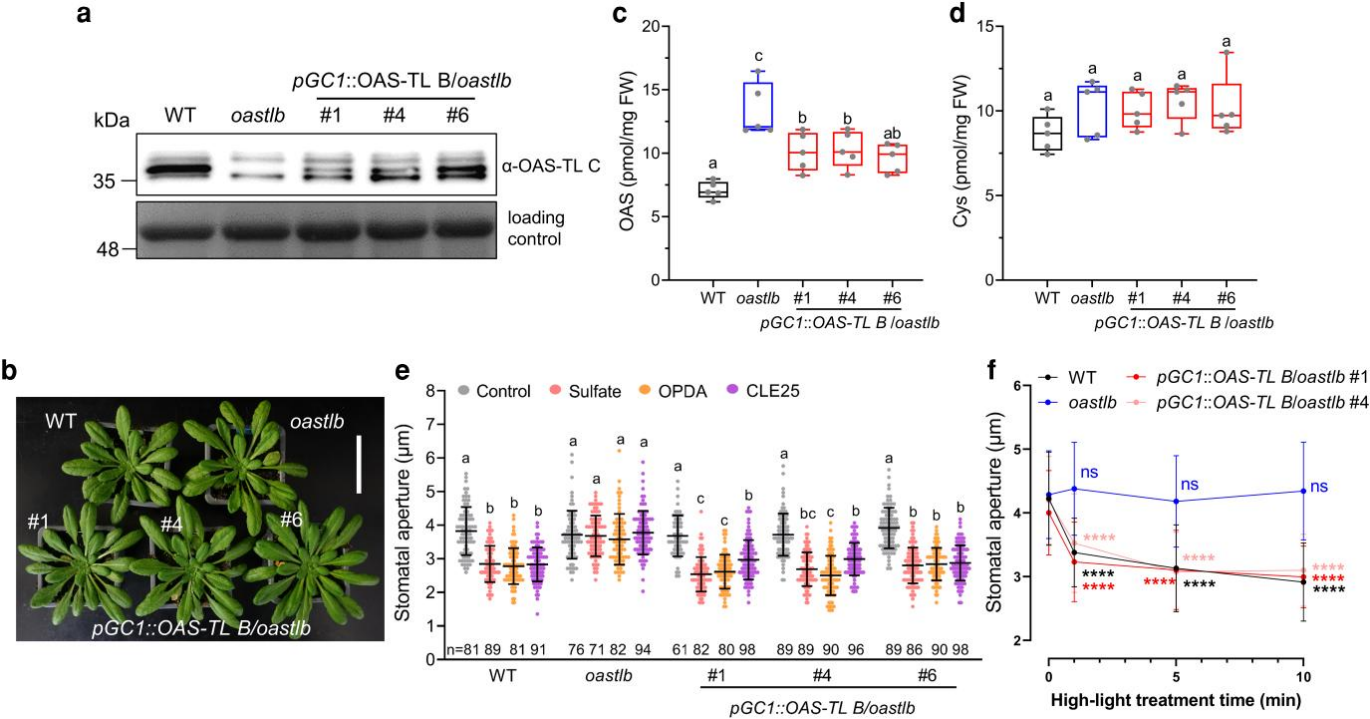
Guard cell-specific complementation of *oastlb* with functional OAS-TL B rescues the loss of stress signals and high-light induced stomata closure. a) Validation of OAS-TL B expression in leaves of three independent *pGC1*::*OAS-TL B oastlb* lines by immunoblot analysis. OAS-TL B protein (middle signal) is detected by an antibody raised against AtOAS-TL C (α-OAS-TL C), which also detects other OAS-TL isoforms. Amido black staining of the Rubisco large subunit served as a loading control. b) Growth phenotype of wild type (WT, Col-0), *oastlb* and *pGC1*::*OAS-TL B oastlb* complemented lines grown on soil for 8 weeks under short day conditions. Scale bar, 5 cm. c) and d) OAS and Cys concentrations in leaves of WT, *oastlb* and *pGC1*::*OAS-TL B oastlb* complemented lines grown on soil under short day conditions for 7 weeks (*n* = 5). e) Apertures of stomata embedded in epidermal peels of WT, *oastlb* and the *pGC1*::*OAS-TL B oastlb* complemented lines treated with sulfate, OPDA or CLE25 for 3 h (*n* = 61-98). f) Time course of stomatal aperture response to high-light in wild type (WT), *oastlb* and guard cell-specific complementation lines. Apertures of imprinted stomata from leaves of WT, *oastlb* and two *pGC1*::*OAS-TL B oastlb* lines after high-light treatment (2,000 µmol m^−2^ s^−1^) for 0, 1, 5, or 10 min (*n* = 150). Data in c) and d) are shown as boxplot. The box plots display medians (horizontal lines), 25% to 75% ranges (boxes) and min to max (whiskers). Data in e) and f) are shown as mean ± SD. Data in c-e) were analysed by one-way ANOVA followed by Tukey's test. Different letters indicate significant differences among different genotypes (*P* < 0.05). Data in f) were analyzed by two-way ANOVA followed by Tukey's test. Statistical differences compared with the control (time point 0) are indicated. ****P < 0.0001. ns, no significant difference.

High-light stress-induced stomatal closure is a rapid physiological response, resulting in significant stomatal closure within 2 min of exposure (Devireddy et al. 2018). In contrast to the wild type, *oastlb* mutants were incapable of stomata closure upon high-light stress for up to 10 min (Figure S4). Reconstruction of the pCSC in guard cells was sufficient to enable the *pGC1::OAS-TL B oastlb* lines to close stomata with indistinguishable kinetics from the wild type (Fig. 1f). High-light stress is known to rapidly induce OPDA accumulation, SERAT activity, and a transcriptional response that depends on the COPS module, which consists of the stromal proteins pCSC, cyclophilin 20-3, and 2-Cys-peroxiredoxin A/B (Speiser et al. 2015; Müller et al. 2017). It is worth noting that OPDA also accumulates under drought, whereas JA is not affected (Savchenko et al. 2014). Thus, it is possible that OPDA even contributes to drought-induced stomatal closure. A signaling function of OPDA in stomata closure is further promoted by the recent findings that the transcription factor AtMYB60 regulates stomatal movement by modulating oxylipin synthesis in guard cells (Simeoni et al. 2022) and that OPDA inhibits blue-light-triggered stomatal opening (Chang et al. 2023).

Sulfate, CLE25, and OPDA trigger pCSC formation to stimulate OAS synthesis for cysteine production, but OAS dissociates the pCSC as part of the negative feedback loop controlling cysteine biosynthesis (Wirtz and Hell 2006). To overcome this negative feedback loop, we genetically engineered an enzymatically inactive OAS-TL B(M167A) protein mutant that, when combined with SERAT2;1, forms an OAS-dissociation-resistant pCSC (Sun et al. 2025). We expressed *OAS-TL B(M167A)* under control of the *GC1* promoter in the wild type to generate plants with a constitutively activated pCSC in guard cells (Figure S5). In the rice mutant *astol1*, formation of an OAS-dissociation-resistant pCSC in all cells causes substantial accumulation of OAS and cysteine but impairs growth due to overproduction of cysteine (Sun et al. 2021). However, the *pGC1*::*OAS-TL B(M167A)* lines were neither retarded in growth, nor accumulated increased levels of OAS and cysteine (Fig. 2a–c). The apparent discrepancy may be explained by the fact that guard cells represent < 4% of the leaf volume (Tolleter et al. 2024), or by the hypothesis that the pCSC in Arabidopsis has a different function than the pCSC in rice. Nevertheless, expression of OAS-TL B(M167A) in guard cells caused significant stomatal closure and upregulation of ABA-responsive genes in epidermal peels that are enriched in guard cells when compared with leaves. We infer from this indirect evidence that guard cell-specific cysteine biosynthesis induced by pCSC formation is sufficient to trigger stomata closure (Fig. 2d, e, and Figure S6). In order to demonstrate that ABA from surrounding cells is not required for stomata closure by a constitutively activated pCSC, we crossed the *p35S*::*OAS-TL B(M167A)* with the *abcg40-2* line, which is deficient in ABA import into guard cells (Sun et al. 2025). The constitutively closed stomata phenotype of p*35S*::*OAS-TL B(M167A)* was not affected by eliminating ABA import into guard cells in the double mutant, strongly suggesting that ABA production in the vasculature is not critical for the constitutively closed stomata phenotype of plants expressing an OAS-dissociation-resistant pCSC (Figure S7).

**Figure 2:**
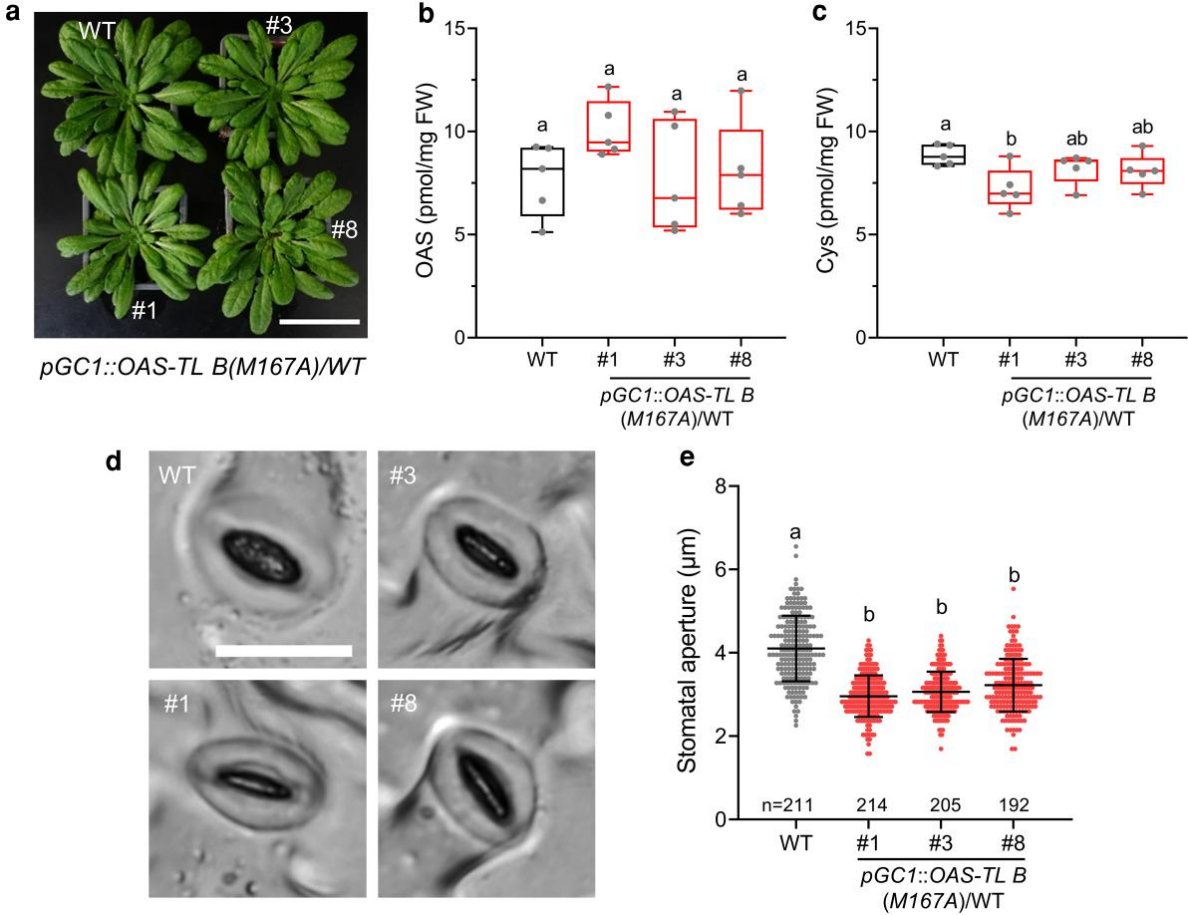
Guard cell-specific activation of pCSC causes stomatal closure in *Arabidopsis*. a) Growth phenotype of wild type (WT, Col-0) and *pGC1*::*OAS-TL B(M167A)* transgenic plants grown on soil for 9 weeks under short day conditions. Scale bar, 5 cm. b) and c) OAS and Cys concentrations in leaves of WT and *pGC1*::*OAS-TL B(M167A)* transgenic plants grown on soil under short day conditions for 7 weeks (*n* = 5). d) Representative images of imprinted stomata from 7-week-old WT and *pGC1*::*OAS-TL B(M167A)* transgenic plants. Scale bar applies to all images, 20 µm. e) Apertures of imprinted stomata from leaves of 7-week-old WT and *pGC1*::*OAS-TL B(M167A)* transgenic plants (*n* = 192 to 214). Data in b) and c) are shown as boxplot. The box plots display medians (horizontal lines), 25% to 75% ranges (boxes) and min to max (whiskers). Data in e are shown as mean ± SD. Data were analysed by one-way ANOVA followed by Tukey's test. Different letters indicate significant differences among different genotypes (*P* < 0.05).

In summary, our data suggest that pCSC assembly in the guard cells is required and sufficient to trigger stomatal closure in response to the soil-drying signals, sulfate and CLE25, and the high-light signal OPDA. These findings strongly suggest that guard cell-autonomous de novo ABA biosynthesis is not only sufficient for stomatal closure in response to decreased air humidity (Bauer et al. 2013), but also for stomatal closure induced by soil drying and high-light stress.

## Supplementary Material

kiag263_Supplementary_Data

## Data Availability

The data underlying this article are available in the article and in its online Supplementary material.
