# Supplementary material for: The chloroplast cysteine synthase complex in guard cells is critical for stress-induced stomatal closure
Source: Plant Physiol. 2026 May 18;201(1):kiag263. doi: 10.1093/plphys/kiag263 (PMC13181391; doi:10.1093/plphys/kiag263)
Supplement: kiag263_Supplementary_Data [file kiag263_supplementary_data.pdf]

Supplementary Materials and Methods

Plant material and growth conditions

In this study, we used Arabidopsis (*Arabidopsis thaliana*) ecotype Col-0 as the wild type and the OAS-TL B loss-of-function mutant (*oastlb*, SALK_021183) described in (Heeg et al., 2008). Plants were grown on soil under short-day conditions (8.5 h light, 100 µmol m^{-2} s^{-1} at 22 ºC and 15.5 h dark at 18 ºC) for all physiological experiments. For the generation of seeds and transgenic plants, the respective genotypes were transferred after week 10 to long-day conditions (16 h light, 100 µmol m-2 s-1 at 22 ºC and 8 h dark at 18 ºC).

Plasmid construction and transformation of plants

The *pGC1::mVenus*, *pGC1::OAS-TL B* and *pGC1::OAS-TL B(M167A)* constructs were generated by the GreenGate method (Lampropoulos et al., 2013), and primers were defined in Supplementary Table 1. In brief, the open reading frames (ORFs) of OAS-TL B variants were PCR-amplified and cloned into the pGGC module vector using *BsaI* sites. Different A-F modules (pGGA-pGC1(AT1G22690), pGGB-empty, pGGC-mVenus or pGGC-OAS-TL B or pGGC-OAS-TL B(M167A), pGGD-empty, pGGE-35S terminator, pGGF-Basta) were fused and then inserted into the pGGZ0003 vector via GreenGate cloning. The resulting plasmids were transferred into the *Agrobacteria* strain GV3101(pSoup), which was then used to generate transgenic plants via *Agrobacterium*-mediated floral dip transformation. Soil-grown transgenic plants were screened seven days after germination by spraying Basta™ (Bayer Crop Science, 1:1000 dilution).

Plant protein extraction and immunoblotting analysis

Total soluble proteins were extracted from plant leaves with 500 µl protein extraction buffer (50 mM Hepes / KOH pH 7.4, 10 mM KCl, 1 mM EDTA, 1 mM EGTA, 10 mM DTT) supplemented with 1× protease inhibitor cocktail (Roche). After being mixed with Laemmli sample buffer (2 % SDS, 4 % glycerine, 20 mM Tris, 0.02 % bromphenol blue, 5 % β-mercaptoethanol, pH 7) and denatured by heating (95 ºC, 10 min), proteins were separated by SDS-Polyacrylamide gel-electrophoresis and detected by immunoblotting using a rabbit anti-OAS-TL C antibody (1:2000 dilution, Heeg et al., 2008), and an HPR-conjugated goat anti-rabbit IgG (AS10852, Agrisera, 1:20000 dilution). The secondary antibody was visualized using the WesternBright™ chemiluminescent substrate (Biozym), and the signals were detected and recorded with the Azure 300 gel imaging system (Azure Biosystems).

Quantification of stomatal aperture

Effector treatments and quantification of stomatal aperture were performed as described in Sun et al. (2025). In brief, epidermal peels were obtained from the abaxial side of 7-week-old Arabidopsis leaves (from at least 3 individual plants) and floated on stomata opening buffer (50 mM KCl, 10 mM MES, pH 5.5) under constant light for 2 h. After washing with distilled water (pH 5.5) for 5 min, the peels were transferred to distilled water (pH 5.5, control) or an aqueous solution containing the effectors (15 mM $MgSO_4$, 10 µM OPDA (Cayman chemical), 1 µM CLE25 peptide (RKVPNGPDPIHN)) for different times (0, 0.5, 1, 2, 3 h). Stomata were imaged with the Leica DMIRB microscope. The width of the stomatal aperture was calculated with ImageJ (version 1.52a). For high-light treatment, plant leaves were exposed to high-light (2000 µmol m^{-2} s^{-1}) at 22 °C for up to 10 min using a halogen cold light source (PL3000, Photonic). For the measurements of stomatal aperture from leaves of non-treated plants and plants treated with high-light, the abaxial side of leaves was imprinted with superglue liquid (UHU). The imprinted stomata were imaged with a Leica DMIRB microscope, and the stomata aperture was determined with ImageJ (version 1.52a). The quantification position of the stomata aperture is located at the width of the centre of the pore as described previously (Sun et al., 2025). Stomata imaging and measurement were performed in a double-blinded manner to avoid any bias during the analysis.

Metabolites analysis

OAS and thiols were detected in the leaves of 7-week-old plants that were snap-frozen and ground in liquid nitrogen. Metabolites were extracted from around 50 mg plant tissue in 300 µl of 0.1 M HCl. After centrifugation, 50 µl and 25 µl of the supernatant were derivatized with AccQ-tag (for OAS measurement) and monobromobimane (mBB, for thiols measurement), respectively, and then quantified by reverse-phase ultra-performance liquid chromatography (UPLC, Waters) using an external standard curve as described previously (Wirtz et al., 2004).

RNA extraction and quantitative real-time PCR

Total RNA was extracted from epidermal peels using a Universal RNA purification kit (EURx) following the manufacturer's protocol. First-strand cDNA was synthesized from RNA using FastGene Scriptase II cDNA Kit (Nippon Genetics) according to the manufacturer's protocol. Quantitative real-time PCR (RT-qPCR) was performed on a Rotor-Gene Q cycler (Qiagen) using a qPCRBIO SyGreen Mix Lo-ROX (PCR Biosystems) according to the manufacturer's instructions. The Arabidopsis *PP2A* gene (AT1G13320) was used as the reference gene. The

expression level of each gene was calculated as $2^{-\Delta\Delta Ct}$ relative to the reference gene. The primers used for RT-qPCR are listed in Supplementary Table 1.

Statistical analyses

Unless otherwise stated, the number of samples is indicated as the n value in figure legends. The statistical analyses were performed in Prism 9.0 (Graphpad). Normal distribution was tested with Shapiro-Wilk. Data sets in different columns were analyzed for statistical significance with the one-way ANOVA, followed by Tukey's test for multiple comparisons. Letters or asterisks indicate significant difference in the figures. In the case of multiple comparisons of grouped (two factors) analyses, the two-way ANOVA followed by Tukey's test was applied (only in Figure 1F and Supplementary Figure S3).

Reference

Heeg C, Kruse C, Jost R, Gutensohn M, Ruppert T, Wirtz M, Hell R (2008) Analysis of the Arabidopsis O-acetylserine(thiol)lyase gene family demonstrates compartment-specific differences in the regulation of cysteine synthesis. Plant Cell **20:** 168-185

Sun SK, Ahmad N, Callenius H, Rajab H, Uslu VV, Cruz Cruz JR, Zhao FJ, Wirtz M, Hell R (2025) The plastid cysteine synthase complex regulates ABA biosynthesis and stomatal closure in Arabidopsis. Nat Commun **16:** 8960

Wirtz M, Droux M, Hell R (2004) O-acetylserine (thiol) lyase: an enigmatic enzyme of plant cysteine biosynthesis revisited in Arabidopsis thaliana. J Exp Bot **55:** 1785-1798

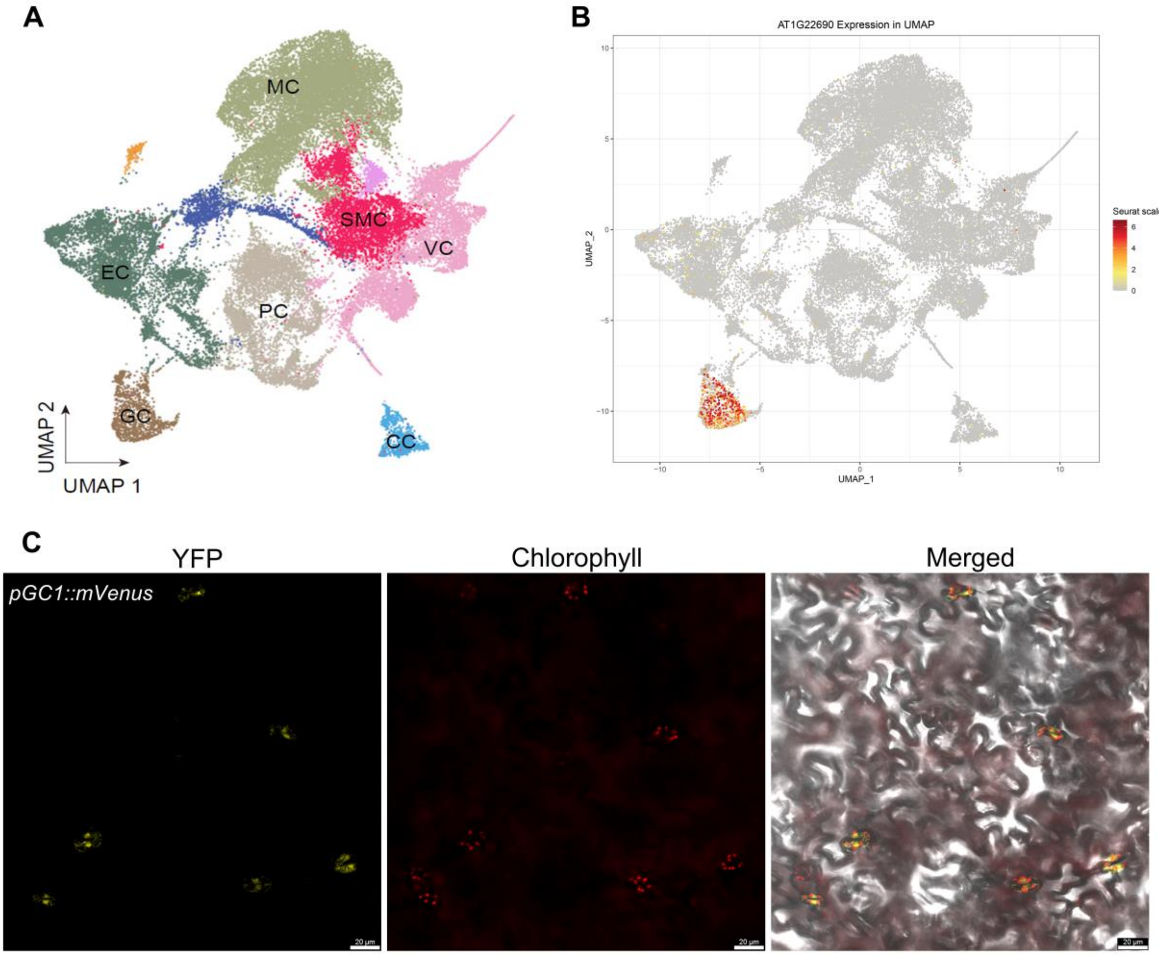

Supplementary Figure S1. Guard cell-specific expression driven by the GC1 promoter is confirmed by transcriptome profiling of single cells and by plant transformation.

A) Standard visualization of cell type using the UMAP method according to a single-cell analysis database (Zhang *et al.* 2021). GC, guard cell. EC, epidermal cell. MC, mesophyll cell. SMC, shoot meristematic cell. PC, proliferating cell. VC, vascular cell. CC, companion cell. **B**) Single-cell gene expression of *GC1* (AT1G22690) according to the published database. The expression of genes is visualized with the Arabidopsis Shoot Apex Cell Atlas database (http://wanglab.sippe.ac.cn/shootatlas/). **C**) Representative images of transgenic plants expressing *pGC1::mVenus*. YFP fluorescence (yellow) and chlorophyll autofluorescence (red) of the leaf abaxial side of 4-week-old *pGC1::mVenus* plant indicates that the *GC1* promoter drives gene expression specifically in guard cells. Similar pattern images are obtained from multiple *pGC1::mVenus* plants. Scale bars, 20 µm.

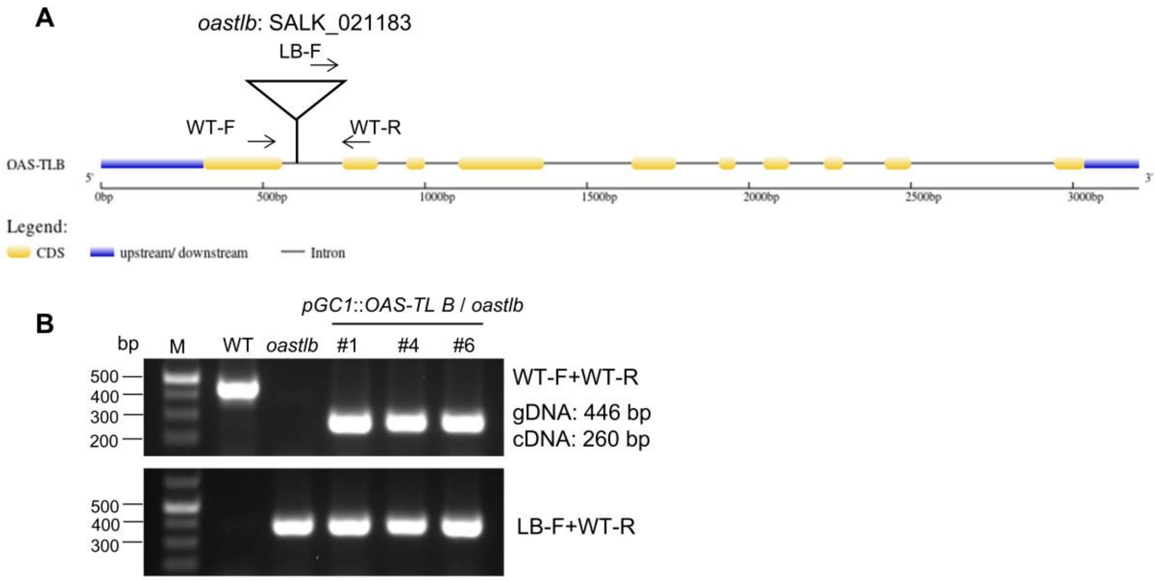

Supplementary Figure S2. Genotyping of positive guard cell-specific complementation lines.

A) Schematic representation of the OAS-TL B gene structure and the insertion site of the T-DNA in the *oastlb* knockout line (SALK_021183). Exons are indicated as yellow boxes. Introns are indicated as black lines. Untranslated regions are indicated as blue boxes. Primers for the genetic characterization are indicated by small black arrows and defined in Supplementary table 1. **B**) Genomic characterization of positive guard cell-specific complementation lines in the *oastlb* background. Genomic DNA was used as the template and amplified with respective primers (WT-F + WT-R for the wild type allele with a size of 446 bp and LB-F+WT-R for the T-DNA insertion ~390 bp). Complementation of *oastlb* with OAS-TLB driven by a guard cell-specific promoter produces an additional signal (cDNA: 260 bp) when the primer pair WT-F + WT-R is used.

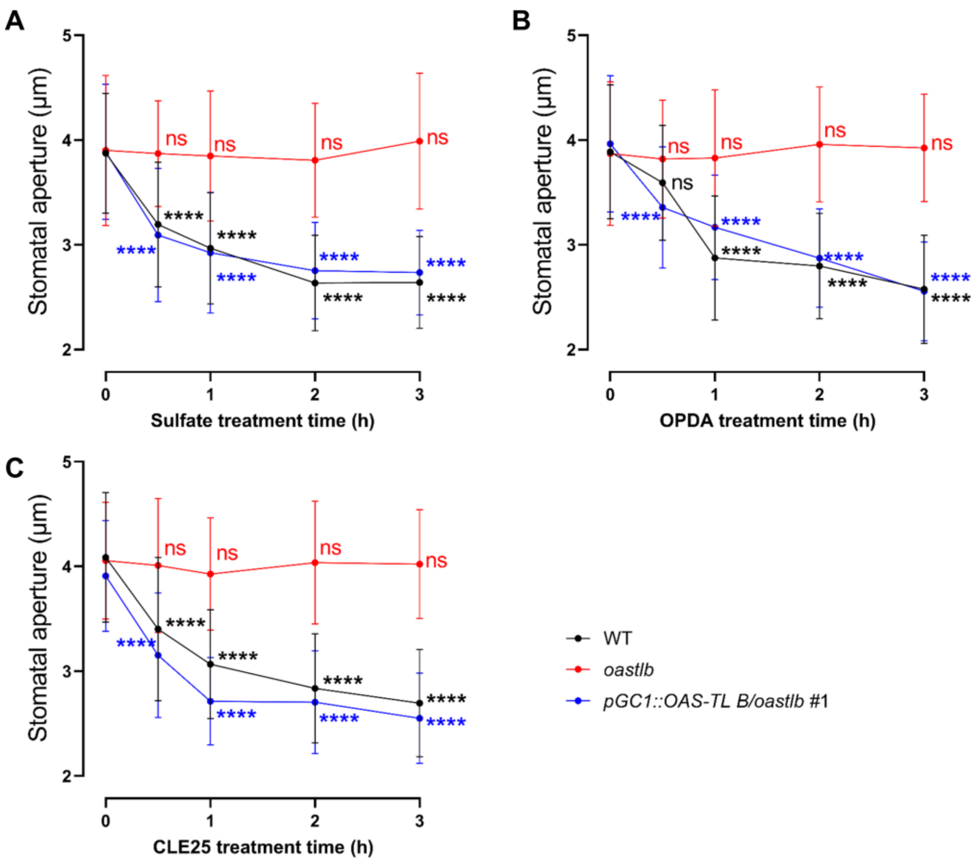

Supplementary Figure S3. Time-resolved analysis of the impact of the signals sulfate, OPDA, and CLE25 on stomata closure in the wild type (WT), *oastlb*, and the *pGC1::OAS-TL B*/*oastlb* line.

A-**C**) Stomatal aperture was determined after application of water (control), 15 mM sulfate (**A**), 10 µM OPDA (**B**), or the peptide hormone CLE25 (1µM, **C**) for up to three hours (n = 50). Data in **A**-**C** are shown as mean ± SD. Statistical differences were analyzed by two-way ANOVA followed by Tukey's test, compared to the relative control (time point 0). ****$P < 0.0001$. ns, no significant difference.

A

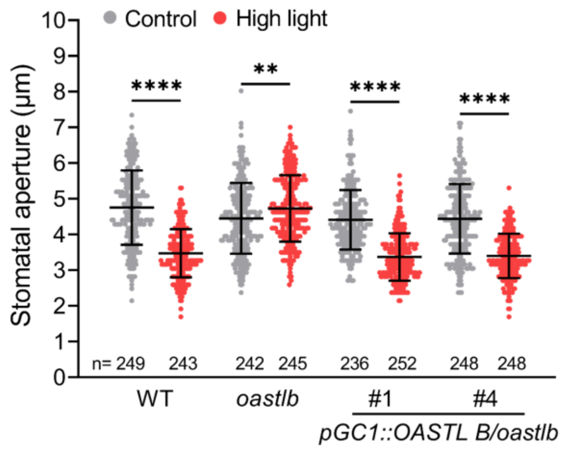

Supplementary Figure S4. Impact of high-light on stomatal aperture in the wild type (WT), *oastlb*, and the *pGC1::OAS-TLB*/*oastlb* lines.

A) Apertures of imprinted stomata from leaves of WT, *oastlb* and the *pGC1::OAS-TL B/oastlb* complemented lines after high-light treatment for 10 min (n =236-252). Data are shown as mean ± SD. Statistical differences were analyzed by one-way ANOVA followed by Tukey's test. ***P* < 0.01. *****P* < 0.0001.

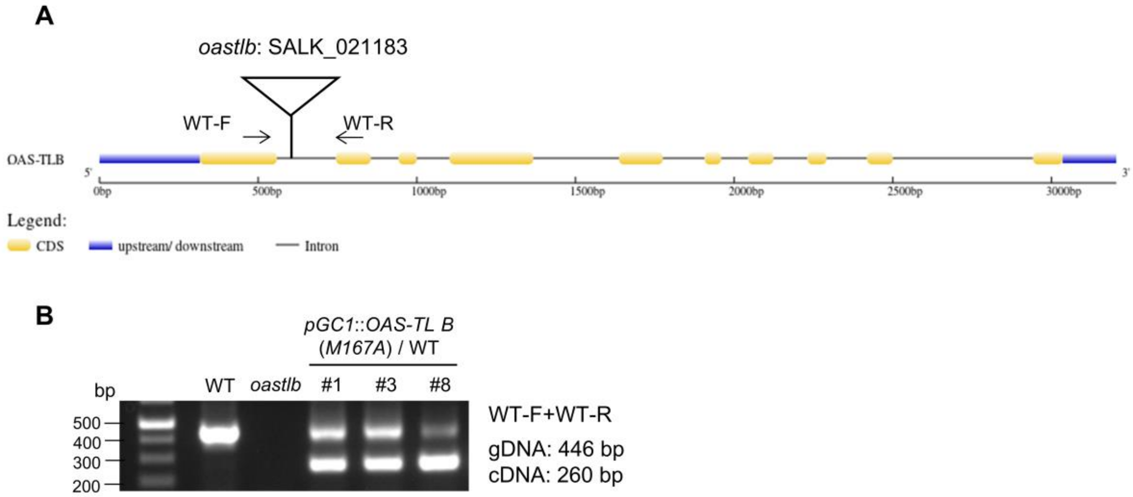

Supplementary Figure S5. Genotyping of wild type plants expressing the OAS-TLB (M167A) protein in guard cells.

A) Structure of the OAS-TL B gene and insertion site of T-DNA in *oastlb* knockout line (SALK_021183) are shown. Exons are indicated as yellow boxes. Introns are indicated as black lines. Untranslated regions are indicated as blue boxes. Primers for the genetic characterization are indicated by small black arrows. Primers for the genetic characterization are indicated by small black arrows and defined in Supplementary table 1. **B**) Genomic characterization of positive guard cell-specific complementation lines in the wild type background. Genomic DNA was used as the template and amplified with respective primers (WT-F + WT-R for the wild type allele with a size of 446 bp). Expression of OAS-TLB(M167A) driven from a guard cell-specific promoter causes an additional signal (cDNA: 260 bp) when the primer pair WT-F + WT-R was applied.

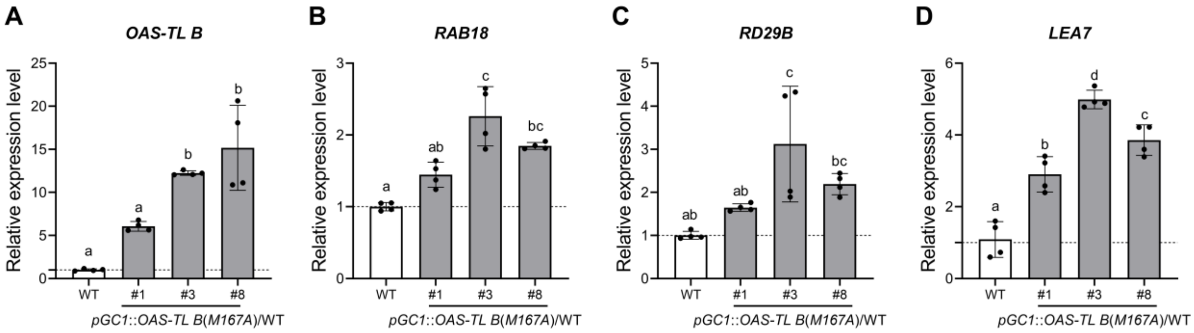

Supplementary Figure S6. Relative expression of *OAS-TL B* and ABA response genes in epidermal peels of wild type (WT) and *pGC1::OAS-TL B* (*M167A*) transgenic plants. **A-D**) Epidermal peels were collected from 7-week-old plants and incubated on water for 2 h. RNA was extracted and the steady-state transcript levels of *OAS-TL B* and ABA-response genes (*RAB18*, *RD29B*, and *LEA7*) were quantified by RT-qPCR. The transcript levels of respective genes from WT samples were set to 1. Data are shown as mean ± SD (n = 4). Data were analysed by one-way ANOVA followed by Tukey's test. Different letters indicate significant differences among different genotypes (P <0.05).

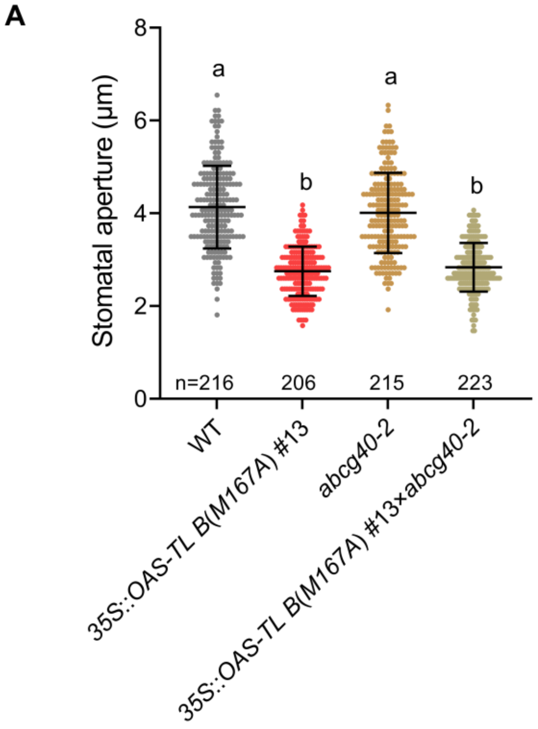

Supplementary Figure S7. Constitutively activated pCSC induced stomatal closure is independent of ABCG40-mediated ABA transport into guard cells. **A**) Apertures of imprinted stomata from leaves of WT, *abcg40-2*, and transgenic plants expressing *OAS-TL B*(*M167A*) in the WT or *abcg40-2* background. Data are shown as mean ± SD (n = 206-223). Data were analysed by one-way ANOVA followed by Tukey's test. Different letters indicate significant differences among different genotypes (P <0.05).

Supplementary Table 1 Primers used for plasmid construction and analysis.

Primer Name	Forward oligonucleotide (5'-3')	Reverse oligonucleotide (5'-3')	Purpose
OAS-TLB-CDS	ATGGCGGCGACATCTTCCTC	TCAAAGCTCGGGCTGCATTTG	To PCR amplify CDS regeion of OAS-TL B
OAS-TLB (M167A)	CTTATCTTGACGGCGCCTGCGTC	GACGCAGGCGCCGTCAAGATAAG	To point mutation
OAS-TLB-pGGC	AACAGGTCTCAGGCTGTATGGCGGCGACATCTTCCTC	AACAGGTCTCACTGATCAAAGCTCGGGCTGCATTTG	To clone OAS-TL B and OAS-TL B(M167A) into green gate module C
WT-F + WT-R	TGACTTCTCGCCACCGTCCT	TGCAACACAGCCCTTGACTACA	genotyping
LB-F + WT-R	ATTTTGCCGATTTCGGAAC	TGCAACACAGCCCTTGACTACA	genotyping
PP2A-qPCR	CTTCTCGCTCCAGTAATGGGATCC	GCTTGGTCGACTATCGGAATGAGAG	RT-qPCR
OAS-TL B-qPCR	GTGTCGAACCCACGGAAAGT	ACCAACAAGCCTTCCTGGAG	RT-qPCR
RAB18-qPCR	GAGCAACTCCACAAGGAAAGTG	ATCATGATGACCTGGCAACTTC	RT-qPCR
RD29B-qPCR	CAGAGAAGCTGAAACCTGGAGA	ATCTTCTCAACCGTCACTTCCA	RT-qPCR
LEA7-qPCR	CAAGAACAGAGTTACAAAGCTGG	ACTGGGCTGTCTCTTGAGTTTT	RT-qPCR
